# Detecting shielded explosives by coupling prompt gamma neutron activation analysis and deep neural networks

**DOI:** 10.1038/s41598-020-70537-6

**Published:** 2020-08-10

**Authors:** K. Hossny, Ahmad Hany Hossny, S. Magdi, Abdelfattah Y. Soliman, Mohammed Hossny

**Affiliations:** 1 British University in Egypt, Cairo, Egypt; 2 OpenAnalytics.ai, Melbourne, Australia; 3 Alexandria University, Alexandria, Egypt; 4 King Abdulaziz University, Jeddah, Saudi Arabia; 5 Deakin University, Geelong, Australia

**Keywords:** Physics, Techniques and instrumentation, Characterization and analytical techniques

## Abstract

Prompt Gamma Neutron Activation Analysis is a nuclear-based technique that can be used in explosives detection. It relies on bombarding unknown samples with neutrons emitted from a neutron source. These neutrons interact with the sample nuclei emitting the gamma spectrum with peaks at specific energies, which are considered a fingerprint for the sample composition. Analyzing these peaks heights will give information about the unknown sample material composition. Shielding the sample from gamma rays or neutrons will affect the gamma spectrum obtained to be analyzed, providing a false indication about the sample constituents, especially when the shield is unknown. Here we show how using deep neural networks can solve the shielding drawback associated with the prompt gamma neutron activation analysis technique in explosives detection. We found that the introduced end-to-end framework was capable of differentiating between explosive and non-explosive hydrocarbons with accuracy of 95% for the previously included explosives in the model development data set. It was also, capable of generalizing with accuracy 80% over the explosives which were not included in the model development data set. Our results show that coupling prompt gamma neutron activation analysis with deep neural networks has a good potential for high accuracy explosives detection regardless of the shield presence.

## Introduction

Explosives detection^1–4^ has been an open-end problem since World War I (WWI)^5^. Due to the recent technological advancements and intelligence of organized terrorist groups around the globe, they were capable of hacking lots of traditional explosives detection techniques. That being said, researchers have been working on alternative explosives detection systems that can outperform conventional methods relying on sniffing dogs and X-ray machines^6^. Hence, many researchers have been paying attention to alternative techniques in explosives detection. Most of the research was focusing on chemically detecting explosives such as using chemically modified multiplexed mode with nanoelectrical devices arrays as a method for super sensitive explosives identification and discrimination^7^. Others were capable of identifying dinitrotoluene at room temperature using a reduced graphene-based oxide gas sensor when modified with a peptide receptor^8^. Research effort demonstrated the capabilities of microporous polymer networks as easily and low-cost manufactural devices for explosives detection^9^. Appreciating the huge analytical power of machine learning in clustering, regressing, and classification of data, some research has been conducted in using various machine learning techniques in analyzing sensors’ data for explosives detection in different environments. Some researchers worked on visualizing explosives by three-dimensional voxel radar using convolutional neural networks^10^. In addition, Deep learning was implemented in detecting explosives using handheld ground penetrating radar (GPR)^11^. Multilayer perceptron models (MLPs) from the artificial neural networks (ANNs) family of artificial intelligence was coupled with pulsed fast thermal neutron activation (PFTNA) technique for detecting explosives^12^. It also showed an accuracy of 97% in forecasting the presence of explosives and drugs when coupled with images obtained from thermal neutrons tomography^13^.

**Figure 1:**
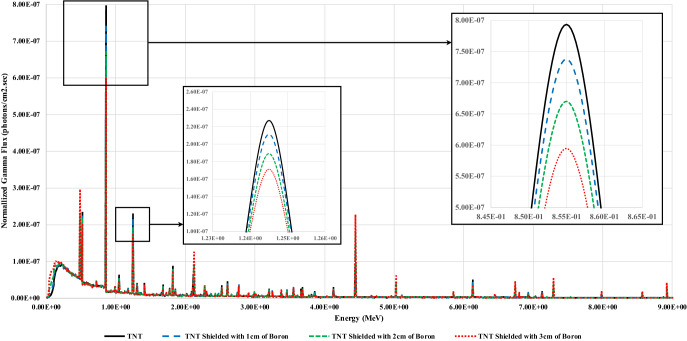
Sample of PGNAA obtained gamma spectra for TNT when shielded with boron.

Nuclear based techniques for explosives detection were introduced in 1986^4^. In the past 20 years, lots of researchers focused on using those techniques in explosives detection for aviation security purposes^14^. Prompt gamma neutron activation analysis (PGNAA) was studied extensively due to its vast potential and applicability. PGNAA is a quantitative isotopic identification technique. A PGNAA system broadly consists of a neutrons source, unknown sample (target to be investigated), and a detector array^15^. When the target is bombarded with the neutron beam, neutrons interact with the target nuclei, emitting the gamma spectrum that includes peaks at certain energies^3^. These energies represent the fingerprints of the target isotopic composition. Analyzing the heights of the peaks emitted at each energy yields the quantitative information about the sample material composition^16^. One of the main advantages of PGNAA in explosives detection is that the irradiation and detection process occur simultaneously^17^. Hence, PGNAA showed extremely high efficiency in identifying explosives, along with reducing the time needed for luggage investigation in airports and on borders. This will reduce the delay time in the passengers' queue^18^. PGNAA has one major drawback, which is shielding the target to be investigated^19,20^. Once the target is shielded, whether the shield is for neutrons or gamma rays, the shield distorts the gamma spectrum read by the detectors, as illustrated in Fig. 1. Hence, the system will not be able to recognize the peak heights correctly, resulting in the false prediction of the target isotopic composition. One other drawback of using PGNAA in explosives detection is the need for a skilled operator to build a decision based on the system’s results^21^. Using machine learning regressors and classifiers such as K-nearest neighbor (KNN) regressors and decision tree classifier to analyze the gamma spectra resulted in 92% accuracy in differentiating between explosive and non-explosive hydrocarbons^22^.

The shielding issue was discussed in multiple studies. Some researchers focused on the shield thickness, and others focused on studying the neutron shield effect on the explosives detection capabilities^23–26^. In this article, we show how coupling deep neural networks with the PGNAA technique can significantly help to solve the shielding issue. This coupling will result in an end-to-end automated framework that will reduce the need for a skilled operator to analyze the gamma spectra read by the detectors array.

**Figure 2:**
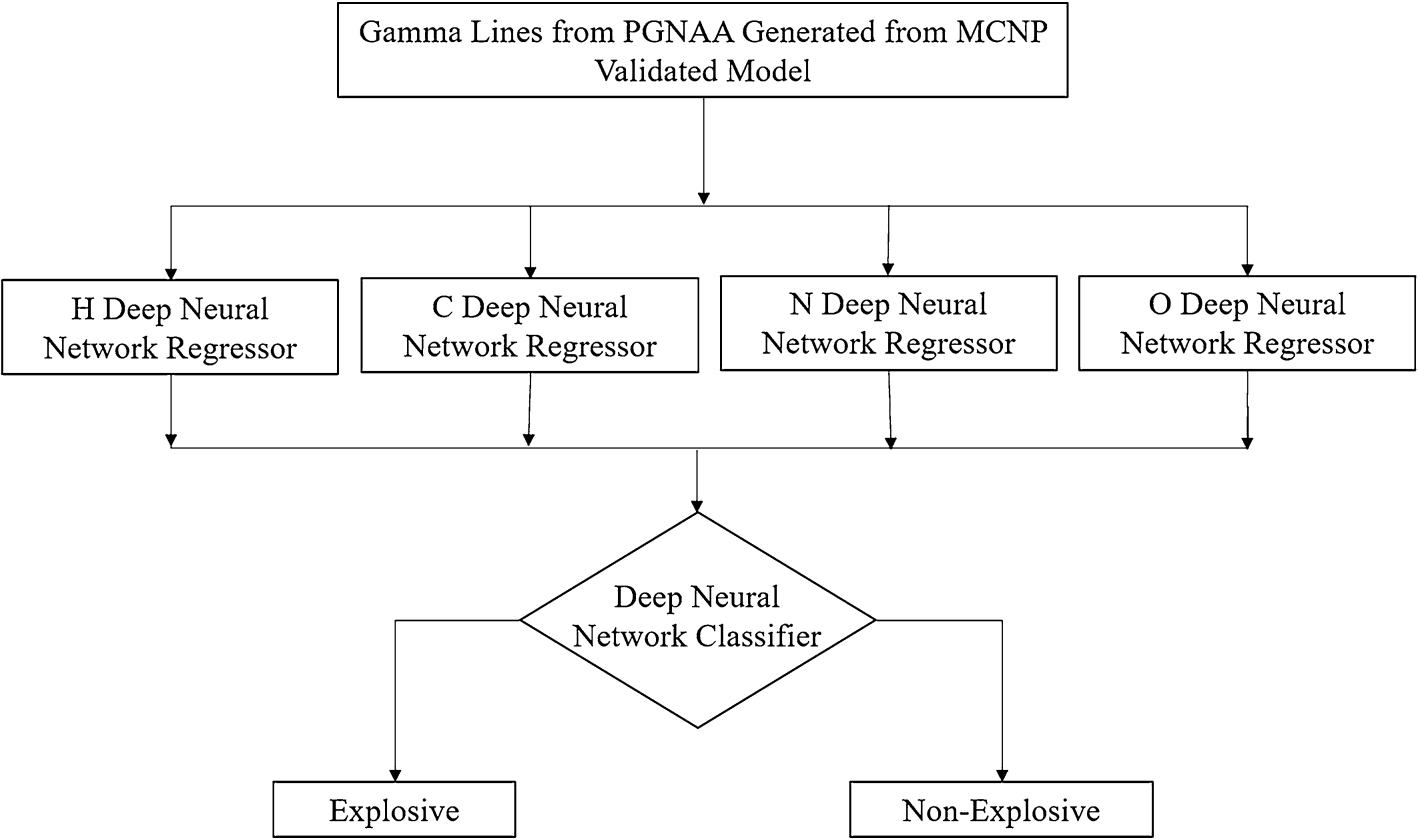
Proposed end-to-end framework.

In this work, the proposed end-to-end framework consisted of four regressors feeding one classifier. The initial input was gamma energy peaks heights, and the output was whether the combination of those peaks represents a hydrocarbon explosive or not, as illustrated in Fig. 2. The methodology of developing this framework development consisted of three main steps; (1) data generation, (2) regressors development, and (3) classifier development. The framework consisted of a pipeline which is a sequence of data manipulation steps starting with raw data and ending with predicted values with minimal error. These steps include data cleaning, feature selection, feature reduction, building the model, testing the model, tuning the model and predicting the final outcome.

## Data generation

**Figure 3:**
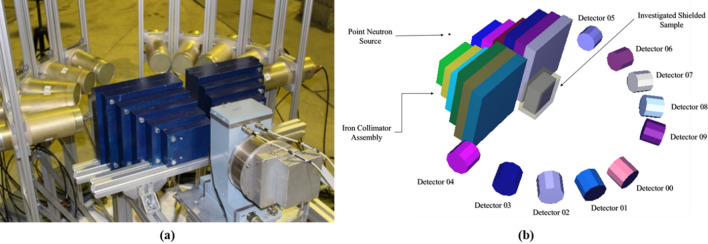
(**a**) Real image of the ROMASHA setup located in Frank laboratory in JINR, (**b**) ROMASHA setup MCNP model developed for data generation.

Due to the sensitivity of the research topic, we used synthesized data instead of experimental data. We used the PGNAA technique in acquiring information about the unknown hydrocarbon sample. Since the proposed framework is developed for differentiating between explosive and non-explosive hydrocarbons regardless of the shield presence. Hence, we focused on the gamma energy peaks representing hydrogen (H), carbon (C), nitrogen (N), and oxygen (O), as listed in Supplementary Table 1^27,28^. For the data generation, we used a Monte Carlo based computational tool for radiation transport calculations (MCNP Code^29^) to mimic the neutron interactions with the samples and gamma spectra read by the detector array^30^. The simulated setup is replicating the Romasha experimental setup located in Frank Laboratory at the Joint Institute for Nuclear Research (JINR) as demonstrated in Fig. 3^31^. The Romasha setup consisted of an ING-27 D-T neutron generator that generates 14.1 meV neutrons, six iron sheets collimator, and Ten BGO detectors located in a semicircle of 30 cm radius, as demonstrated in Fig. 3. Dimensions of the setup are listed in Supplementary Table 2^22,31^. The gamma rays emitted due to neutron interactions with the sample travel in different directions. Hence, using detectors array in PGNAA setups is a standard procedure for detecting the emitted gamma spectrum. We modeled the neutrons emitted from the D-T neutron generator as point isotropic source of 14.1 meV energy. Hence, changing the orientation of the investigated sample will not affect the resulting gamma spectrum. We used our previously developed and validated MCNP model with validation metrics listed in Supplementary Table 3^22^. In the developed MCNP model that we used in the data generation process, we didn’t consider the natural radioactivity background. Natural radioactivity on earth usually includes gamma radiation. The neutron background radiation is insignificant. Hence, it is usually neglected in the application design process. Regarding the gamma background radiation levels, it is a standard procedure for any setup that includes radiation detection, and the detectors are calibrated to remove the background gamma readings from the relevant energy channels. In our case, the relevant channels are listed in Supplementary Table 1. Using the validated MCNP model, we generated 1,478 samples for non-shielded and shielded explosive and non-explosive hydrocarbons with a variation of shield thicknesses from zero (not-shielded) to three cm shield. Twenty-two different hydrocarbon explosives were included in the data generation process, their chemical composition (in mass fractions), densities ($\rho$ in g/cm^3^), volume (*Vol* in cm^3^), and masses (M in g) are listed in Table 1. The shields studied were boron (B), light water (H_2_O), borated light water (BW), polyethylene (Poly), borated polyethylene (BP), lead (Pb), iron (Fe), and steel, the generated data breakdown is listed in Supplementary Table 4. We chose random weight fractions of H, C, N, and O for non-explosive hydrocarbon samples to represent the randomness in hydrocarbon materials compositions that exist in the ordinary luggage to be investigated. To cross-validate the whole pipeline using the leave-one-out method, we divided the generated 1,478 samples into 11 datasets. We separated the final test data set by excluding two explosive hydrocarbons with all of their shield variations (50 samples), and 50 random non-explosive hydrocarbons with random shield variations from each data set. Hence, each development data set consisted of 1,378 samples. Average standard deviation scores associated with the generated data are listed in Supplementary Table 5. In this proof of concept, the obtained gamma spectrum is per source neutron and per second. Also, the gamma spectra input features were normalized between zero and one as a pre-processing measure necessary for the model development stage. Hence, the provided results are for irradiation per second. That being said, we believe that increasing the irradiation time and D-T neutron generator intensity of 10^9^ to 10^12^ n/cm^2^ s during the practical deployment will provide better results. Using a prescreening device such as the X-ray machines will reduce the total screening time by permitting the movement of suspicious baggage to another convoy, which leads to the D-T neutron generator detection system^23^. In practice assuming 10% of the baggage are suspicious and using a high-intensity neutron generator. This will reduce the time of the multibarrier screening process and ensure an efficient detection of both shielded and unshielded illicit materials. The presence of the X-ray prescreening device will also help to direct the neutron generator specific to locations in the parcel and reduce the time for the second screening process^23^.

**Table 1:** Studied explosives chemical compositions.

Explosive	$\rho$ (g/cm^3^)	*V*_*ol*_ (cm^3^)	H	C	N	O	M (g)
Ammonium Picrate	1.72	500	0.025	0.293	0.228	0.455	860
AN	1.72	500	0.050	0.000	0.350	0.600	860
Cl-20	2.04	500	0.014	0.164	0.384	0.438	1,020
DMNB	0.45	500	0.070	0.409	0.159	0.360	225
EGDN	1.49	500	0.026	0.158	0.184	0.632	745
Guanidine nitrate	1.44	500	0.050	0.098	0.459	0.393	720
HMTD	1.57	500	0.057	0.346	0.135	0.460	785
HMX	1.91	500	0.027	0.162	0.378	0.432	955
Hydrazine nitrate	1.64	500	0.053	0.000	0.442	0.501	820
Mannitol hexanitrate	1.73	500	0.018	0.159	0.186	0.637	865
NG	1.6	500	0.022	0.159	0.185	0.634	800
NM	1.14	500	0.049	0.200	0.230	0.525	570
PETN	1.77	500	0.025	0.190	0.177	0.607	885
Picric acid	1.76	500	0.013	0.314	0.183	0.489	880
RDX	1.858	500	0.027	0.162	0.378	0.432	929
TATB	1.93	500	0.023	0.279	0.326	0.372	965
TATP	1.2	500	0.081	0.486	0.000	0.432	600
Tetryl	1.73	500	0.017	0.293	0.244	0.446	865
TNAZ	1.84	500	0.021	0.188	0.292	0.500	920
TNB	1.76	500	0.014	0.338	0.197	0.451	880
TNT	1.65	500	0.022	0.370	0.185	0.423	825
UN	1.59	500	0.040	0.097	0.340	0.520	795

## Regressors development

A regressor is a method that generates a model capable of predicting the numerical dependent variable and minimize the error between the predicted value and the actual value for the whole range of the dependent variables and the whole space of the independent variables. We developed four deep neural network regressors to predict the weight fractions of H, C, N, and O in the investigated samples, respectively. The input for each regressor was the 11 gamma energy peak heights read by the ten detectors in the detector array. Hence, the total number of input features was 110 features. Outputs of the four regressors were the H, C, N, and O weight fractions, respectively. Training and test size represented 80% and 20% of each development data set from the data generated, respectively. Hence, we cross-validated each of the developed models across five folds. In each fold, training and test samples were chosen randomly. We used the mean squared error (MSE), mean absolute error (MAE), and the coefficient of determination (R^2^) as the quality metrics for the developed regressors, as listed in Table 2. The R^2^ score is a regression metric that evaluates the quality of fit, and it measures the percentage of the correctly predicted numerical values in comparison to the whole dataset. Although the oxygen weight fraction regressor had the highest test MAE, we considered the regressors responsible for predicting the H, and C elements weight fractions showed the worst and less bad quality metrics as they had the highest differences between training and test MAEs. This indicates a higher tendency to overfit the regression process. The reason behind this is the existence of light water, and polyethylene as shields within the data generated samples. The issue associated with these two particular shields is the existence of H and C within the shield material composition. Putting aside the fact that H and C are considered excellent neutron shields. Neutron interactions with H and C in those shields will also, add to gamma energy peaks’ heights of H, and C read by the detectors array. This will not only distort the resulting gamma spectrum due to neutron shielding, but this will also provide misleading H, and C energy peaks’ heights that do not represent H and C weight fractions in the investigated sample. Although O exists in light water, it is a neutron transparent element, and thus, it has a low probability of interaction with neutrons. That being said, the regressor predicting the O weight fractions showed regression quality metrics better than that of H and C regressors. On the other hand, the regressor responsible for the prediction of N weight fraction showed the least MSE, MAE, and the highest R^2^ scores. This was due to the absence of N in any of the investigated shields.

**Table 2:** Average metrics for the four developed regressors.

Model name	Training MSE	Training MAE	Training R^2^	Test MSE	Test MAE	Test R^2^
Hydrogen	0.001	0.025	0.955	0.003	0.036	0.888
Carbon	0.003	0.038	0.868	0.004	0.043	0.836
Nitrogen	0.002	0.029	0.926	0.002	0.032	0.905
Oxygen	0.004	0.047	0.872	0.004	0.048	0.859

## Classifier development

A classifier is a method that builds a model capable of identifying different categorical data items according to the set of features associated with them. In the last stage of our pipeline, we developed a classifier to differentiate between explosive and non-explosive hydrocarbons regardless of whether the investigated hydrocarbon was shielded or not. The input for the classifier was the output of the four regressors. The classifier’s output was whether the regressed weight fractions of H, C, N, and O represent an explosive or non-explosive hydrocarbon. Similarly to the regressors’ development, training and test data sizes represented 80% and 20% from each development data set from the data generated, respectively. The classifier was also cross-validated over five folds. We used accuracy, precision, recall, and F1 scores as the developed classifier’s quality metrics. Accuracy measures how many of the predicted classes for the categorical values were correctly classified in comparison to the whole data set. The accuracy metric can be misleading if the data was unbalanced. Also, it is not statistically significant. Precision represents the ratio between the predicted true positives and all the positively predicted instances (true positives and false positives). While the recall score is the ratio between the predicted true positive instances and the true number of positives that should have been scored (true positives and false negatives). Finally, F1-score is the harmonic mean for precision and recall. Considering that precision and recall are negatively proportional for most of the models, the high harmonic mean implies a robust model that can predict the true positive, true negative, false positive and false negatives properly. We trained the classifier through feeding the regressed weight fraction values of H, C, N, and O rather than the original values to reduce the error propagation possibility. The developed classifiers showed 95% for all weighted mean quality metrics. Details of each developed classifier quality metrics are listed in Table 3.

**Table 3:** Classification metrics for the 11 developed classifiers.

Model number	Accuracy	Precision	Recall	F1-score
Model 1	0.931	0.931	0.932	0.931
Model 2	0.964	0.964	0.965	0.964
Model 3	0.946	0.946	0.946	0.946
Model 4	0.942	0.942	0.942	0.942
Model 5	0.942	0.942	0.943	0.942
Model 6	0.953	0.953	0.953	0.953
Model 7	0.960	0.960	0.960	0.960
Model 8	0.957	0.957	0.957	0.957
Model 9	0.960	0.960	0.960	0.960
Model 10	0.949	0.950	0.950	0.949
Model 11	0.946	0.946	0.946	0.946
Average	0.950	0.950	0.950	0.950

## Pipeline performance

In summary, we developed 11 pipelines, each pipeline consisted of four regressors to predict the weight fraction values of H, C, N, and O respectively and a classifier to determine whether the investigated sample was an explosive hydrocarbon or not. During the development of each pipeline, one of the 11 development data sets was chosen. Finally, we tested each pipeline twice, once on the development data set, and the other through the corresponding final test data set (data that has not been included in the model development data set neither in training or testing). As expected, testing the pipelines on the development data sets resulted in the same weighted mean accuracy, precision, recall, and F1 scores as that of the classifiers' development scores (95%). On the other hand, when we tested the pipelines on the final test data sets, classification quality metrics’ weighted mean scores dropped to 80%, 79%, 85%, and 80% for the accuracy, precision, recall, and F1 scores respectively. These scores represent the pipeline capability of generalization over unknown explosives and non-explosives regardless of the shield existence or not. Detailed classification metrics scores for the development data set test, and the final test data set test are listed in Tables 4 and 5, respectively. We noticed from the test performed on the final test data sets that the average false alarm rate is 2%.

**Table 4:** Development data set test classification metrics for the 11 developed pipelines.

Model number	Accuracy	Precision	Recall	F1-score
Model 1	0.946	0.947	0.947	0.946
Model 2	0.964	0.964	0.964	0.964
Model 3	0.945	0.945	0.946	0.945
Model 4	0.951	0.952	0.952	0.951
Model 5	0.928	0.929	0.930	0.928
Model 6	0.947	0.947	0.948	0.947
Model 7	0.947	0.947	0.948	0.947
Model 8	0.940	0.940	0.941	0.940
Model 9	0.959	0.959	0.959	0.959
Model 10	0.940	0.940	0.941	0.940
Model 11	0.933	0.934	0.934	0.933
Average	0.946	0.946	0.947	0.946

**Table 5:** Final test data set test classification metrics for the 11 developed pipelines.

Model number	Accuracy	Precision	Recall	F1-score
Model 1	0.810	0.804	0.851	0.810
Model 2	0.650	0.607	0.766	0.650
Model 3	0.680	0.653	0.762	0.680
Model 4	0.760	0.745	0.838	0.760
Model 5	0.850	0.847	0.885	0.850
Model 6	0.840	0.836	0.879	0.840
Model 7	0.950	0.950	0.950	0.950
Model 8	0.730	0.715	0.792	0.730
Model 9	0.720	0.703	0.786	0.720
Model 10	0.860	0.857	0.890	0.860
Model 11	0.930	0.930	0.932	0.930
Average	0.800	0.786	0.848	0.800

## Conclusions

From the above discussion, we concluded that the developed end-to-end framework scored higher classification metrics for previously included explosives in the training process. Due to the nature of security problems, and since there is a finite number of explosives, it is possible to include all the known explosives in the regressors and classifiers training. However, some of the developed pipelines were capable of detecting 920 g of trinitro-azetidine (TNAZ) with accuracy of 84%, 800 g of nitroglycerin (NG) and 825 g of trinitrotoluene (TNT) with accuracy of 88%, 880 g of Picric acid, 865 g of trinitro-phenylmethyl nitramine (tetryl), and 795 g of urea nitrate (UN) with accuracy of 92%, and finally 885 g of pentaerythritol tetranitrate (PETN) with accuracy of 100%. The aforementioned explosives were not included in neither the training nor test of their corresponding development data sets. However, by testing the minimum detectable mass for the PETN across three cm of the studied shields. The pipeline was capable of detecting minimum mass of 708 g of PETN for the shields water, borated water, iron, lead, and boron. Also, it was capable of detecting 177, 354, and 531 g of PETN when shielded with three cm of borated polyethylene, polyethylene, and steel respectively. Thus, coupling deep neural networks with the PGNAA technique showed huge potential in overcoming the neutron and gamma shielding drawback of the PGNAA technique in explosives detection and security applications. We believe that including more massive data sets that include experimental data that includes more parameters can significantly improve the efficiency of the proposed pipeline in explosives detection. Future work may include studying actual luggage with various hydrocarbon compounds placed around the shielded sample (whether it was explosive or not). We used the polyethylene and borated polyethylene shields as they are considered neutron absorbers that can be used in shielding the investigated sample. They also provide insights about the ability to proceed with this work to investigate samples surrounded by items usually placed in ordinary luggage.

## Supplementary information

Supplementary Tables.
