# Supplementary material for: Detecting shielded explosives by coupling prompt gamma neutron activation analysis and deep neural networks
Source: Sci Rep. 2020 Aug 10;10:13467. doi: 10.1038/s41598-020-70537-6 (PMC7417538; doi:10.1038/s41598-020-70537-6)
Supplement: Supplementary file 1 — Supplementary Tables. [file 41598_2020_70537_MOESM1_ESM.docx]

# **Detecting Shielded Explosives by Coupling Prompt Gamma Neutron Activation Analysis and Deep Neural Networks**

**K. Hossny^1*^, Ahmad Hany Hossny^2^, S. Magdi^3^, Abdelfattah Y. Soliman^4^, Mohammed Hossny^5^**

**[1] British University in Egypt, Egypt.**

**[2] Crowdanalytica, Australia.**

**[3] Alexandria University, Egypt.**

**[4] King Abdulaziz University, Saudi Arabia.**

**[5] Deakin University, Australia.**

*** Corresponding Author Email Address: Karim.Hossny@BUE.edu.eg**

| **Supplementary Table 1. Hydrogen, Carbon, Nitrogen, and Oxygen Characteristic Gamma Lines Energies** | | | | | | | | | | | |
| --- | --- | --- | --- | --- | --- | --- | --- | --- | --- | --- | --- |
| **Isotope** | **H** | **C** | **N** | | | **O** | | | | | |
| **Gamma Line Energy (MeV)** | 2.22 | 4.44 | 1.64 | 2.32 | 5.11 | 2.75 | 3.69 | 3.85 | 6.13 | 6.92 | 7.12 |

| **Supplementary Table 2. Romasha Setup Dimensions** | | | | | | |
| --- | --- | --- | --- | --- | --- | --- |
|  | **Layer 1** | **Layer 2** | **Layer 3** | **Layer 4** | **Layer 5** | **Layer 6** |
| **Iron Block Thickness (cm)** | 4 cm | | | | | |
| **Distance Between 2 Opposite Blocks (cm)** | 5.4 | 5.2 | 4.9 | 4.5 | 4.2 | 4 |
| **Blocks Height (cm)** | 23.7 | 23.7 | 22 | 16 | 13 | 10 |
| **Blocks Length (cm)** | 25 | 23.6 | 21.3 | 18.8 | 16.5 | 14 |
| **Distance between Neutron Shield and Iron Shield (cm)** | 9.5 cm | | | | | |
| **Distance between Iron Shield and Sample Center (cm)** | 6.25 cm | | | | | |
| **Distance between Centers of Ch00 and Ch09 (cm)** | 21 cm | | | | | |

| **Supplementary Table 3. MCNP ROMASHA Setup Validation Results for 4.44 MeV Carbon Peak** | | | | |
| --- | --- | --- | --- | --- |
| **Channel** | **Experimental Peak Counts** | **MCNP Model Counts** | **Absolute Error** | **Relative Error (%)** |
| **00** | 360 | 378 | 18 | 5 |
| **01** | 370 | 350 | 20 | 5.4 |
| **02** | 345 | 290 | 55 | 15.9 |
| **03** | 230 | 220 | 10 | 4.3 |
| **04** | 205 | 220 | 15 | 7.3 |
| **05** | 225 | 210 | 15 | 6.6 |
| **06** | 228 | 225 | 3 | 1.3 |
| **07** | 287 | 278 | 9 | 3.1 |
| **08** | 360 | 340 | 20 | 5.5 |
| **09** | 292 | 393 | 101 | 34.5 |

| **Supplementary Table 4. Generated Data Breakdown** | | | | | | | | | |
| --- | --- | --- | --- | --- | --- | --- | --- | --- | --- |
| **Explosive/Shield** | **B** | **H_2_O** | **BW** | **Poly** | **BP** | **Pb** | **Fe** | **Steel** | **No shield** |
| **Ammonium Picrate** | 3 | 3 | 3 | 3 | 3 | 3 | 3 | 3 | 1 |
| **AN** | 3 | 3 | 3 | 3 | 3 | 3 | 3 | 3 | 1 |
| **Cl-20** | 3 | 3 | 3 | 3 | 3 | 3 | 3 | 3 | 1 |
| **DMNB** | 3 | 3 | 3 | 3 | 3 | 3 | 3 | 3 | 1 |
| **EGDN** | 3 | 3 | 3 | 3 | 3 | 3 | 3 | 3 | 1 |
| **Guanidine nitrate** | 3 | 3 | 3 | 3 | 3 | 3 | 3 | 3 | 1 |
| **HMTD** | 3 | 3 | 3 | 3 | 3 | 3 | 3 | 3 | 1 |
| **HMX** | 3 | 3 | 3 | 3 | 3 | 3 | 3 | 3 | 1 |
| **Hydrazine nitrate** | 3 | 3 | 3 | 3 | 3 | 3 | 3 | 3 | 1 |
| **Mannitol Hexanitrate** | 3 | 3 | 3 | 3 | 3 | 3 | 3 | 3 | 1 |
| **NG** | 3 | 3 | 3 | 3 | 3 | 3 | 3 | 3 | 1 |
| **NM** | 3 | 3 | 3 | 3 | 3 | 3 | 3 | 3 | 1 |
| **PETN** | 3 | 3 | 3 | 3 | 3 | 3 | 3 | 3 | 1 |
| **Picric acid** | 3 | 3 | 3 | 3 | 3 | 3 | 3 | 3 | 1 |
| **RDX** | 3 | 3 | 3 | 3 | 3 | 3 | 3 | 3 | 1 |
| **TATB** | 3 | 3 | 3 | 3 | 3 | 3 | 3 | 3 | 1 |
| **TATP** | 3 | 3 | 3 | 3 | 3 | 3 | 3 | 3 | 1 |
| **Tetryl** | 3 | 3 | 3 | 3 | 3 | 3 | 3 | 3 | 1 |
| **TNAZ** | 3 | 3 | 3 | 3 | 3 | 3 | 3 | 3 | 1 |
| **TNB** | 3 | 3 | 3 | 3 | 3 | 3 | 3 | 3 | 1 |
| **TNT** | 3 | 3 | 3 | 3 | 3 | 3 | 3 | 3 | 1 |
| **UN** | 3 | 3 | 3 | 3 | 3 | 3 | 3 | 3 | 1 |
| **Non-Explosive** | 66 | 66 | 66 | 66 | 66 | 66 | 66 | 66 | 400 |
| **Total Number of Samples** | 132 | 132 | 132 | 132 | 132 | 132 | 132 | 132 | 422 |

| **Supplementary Table 5. Average Standard Deviation Scores Associated with the Generated Data for each Detector and Energy Channel.** | | | | | | | | | | |
| --- | --- | --- | --- | --- | --- | --- | --- | --- | --- | --- |
|  | **Channel 00** | | **Channel 01** | | **Channel 02** | | **Channel 03** | | **Channel 04** | |
| **Energy Channel** | **Average** | **STDEV** | **Average** | **STDEV** | **Average** | **STDEV** | **Average** | **STDEV** | **Average** | **STDEV** |
| **1.645** | 2E-10 | 3E-11 | 2E-10 | 3E-11 | 2E-10 | 2E-11 | 2E-10 | 2E-11 | 2E-10 | 2E-11 |
| **2.215** | 2E-10 | 2E-11 | 2E-10 | 2E-11 | 2E-10 | 2E-11 | 2E-10 | 2E-11 | 2E-10 | 2E-11 |
| **2.315** | 2E-10 | 6E-11 | 2E-10 | 6E-11 | 2E-10 | 5E-11 | 2E-10 | 4E-11 | 2E-10 | 5E-11 |
| **2.745** | 3E-10 | 7E-11 | 3E-10 | 7E-11 | 3E-10 | 7E-11 | 3E-10 | 6E-11 | 3E-10 | 6E-11 |
| **3.685** | 5E-10 | 1E-10 | 4E-10 | 1E-10 | 4E-10 | 1E-10 | 4E-10 | 1E-10 | 4E-10 | 1E-10 |
| **3.855** | 3E-10 | 9E-11 | 3E-10 | 8E-11 | 3E-10 | 8E-11 | 3E-10 | 8E-11 | 3E-10 | 7E-11 |
| **4.445** | 6E-10 | 3E-10 | 5E-10 | 3E-10 | 5E-10 | 2E-10 | 5E-10 | 2E-10 | 5E-10 | 2E-10 |
| **5.115** | 2E-10 | 5E-11 | 2E-10 | 5E-11 | 2E-10 | 5E-11 | 2E-10 | 4E-11 | 2E-10 | 3E-11 |
| **6.135** | 6E-10 | 2E-10 | 6E-10 | 2E-10 | 6E-10 | 2E-10 | 5E-10 | 2E-10 | 5E-10 | 2E-10 |
| **6.925** | 3E-10 | 1E-10 | 3E-10 | 1E-10 | 3E-10 | 9E-11 | 3E-10 | 9E-11 | 3E-10 | 8E-11 |
| **7.125** | 3E-10 | 1E-10 | 3E-10 | 1E-10 | 3E-10 | 1E-10 | 3E-10 | 1E-10 | 3E-10 | 1E-10 |
| **Average** | 3E-10 | 1E-10 | 3E-10 | 1E-10 | 3E-10 | 9E-11 | 3E-10 | 8E-11 | 3E-10 | 8E-11 |
| **STDEV** | 1E-10 | 7E-11 | 1E-10 | 7E-11 | 1E-10 | 7E-11 | 1E-10 | 6E-11 | 1E-10 | 6E-11 |
|  | **Detector 05** | | **Detector 06** | | **Detector 07** | | **Detector 08** | | **Detector 09** | |
| **Energy Channel** | **Average** | **STDEV** | **Average** | **STDEV** | **Average** | **STDEV** | **Average** | **STDEV** | **Average** | **STDEV** |
| **1.645** | 2E-10 | 2E-11 | 2E-10 | 2E-11 | 2E-10 | 2E-11 | 2E-10 | 3E-11 | 2E-10 | 3E-11 |
| **2.215** | 2E-10 | 2E-11 | 2E-10 | 2E-11 | 2E-10 | 1E-11 | 2E-10 | 2E-11 | 2E-10 | 2E-11 |
| **2.315** | 2E-10 | 4E-11 | 2E-10 | 4E-11 | 2E-10 | 4E-11 | 2E-10 | 5E-11 | 2E-10 | 6E-11 |
| **2.745** | 3E-10 | 5E-11 | 3E-10 | 7E-11 | 3E-10 | 7E-11 | 3E-10 | 7E-11 | 3E-10 | 7E-11 |
| **3.685** | 4E-10 | 1E-10 | 4E-10 | 1E-10 | 4E-10 | 1E-10 | 4E-10 | 1E-10 | 5E-10 | 1E-10 |
| **3.855** | 3E-10 | 8E-11 | 3E-10 | 7E-11 | 3E-10 | 9E-11 | 3E-10 | 8E-11 | 3E-10 | 8E-11 |
| **4.445** | 5E-10 | 2E-10 | 4E-10 | 2E-10 | 5E-10 | 2E-10 | 5E-10 | 2E-10 | 5E-10 | 3E-10 |
| **5.115** | 1E-10 | 6E-11 | 2E-10 | 5E-11 | 2E-10 | 5E-11 | 2E-10 | 5E-11 | 1E-10 | 5E-11 |
| **6.135** | 5E-10 | 2E-10 | 5E-10 | 2E-10 | 5E-10 | 2E-10 | 6E-10 | 2E-10 | 6E-10 | 2E-10 |
| **6.925** | 3E-10 | 9E-11 | 3E-10 | 8E-11 | 3E-10 | 9E-11 | 3E-10 | 9E-11 | 3E-10 | 8E-11 |
| **7.125** | 3E-10 | 1E-10 | 3E-10 | 1E-10 | 4E-10 | 1E-10 | 3E-10 | 1E-10 | 3E-10 | 1E-10 |
| **Average** | 3E-10 | 9E-11 | 3E-10 | 8E-11 | 3E-10 | 9E-11 | 3E-10 | 1E-10 | 3E-10 | 1E-10 |
| **STDEV** | 1E-10 | 6E-11 | 1E-10 | 6E-11 | 1E-10 | 7E-11 | 1E-10 | 7E-11 | 1E-10 | 7E-11 |
